# Comparative analysis of three- and two-antibody cocktails to AMACR and basal cell markers for the immunohistochemical diagnosis of prostate carcinoma

**DOI:** 10.1186/1746-1596-7-81

**Published:** 2012-07-16

**Authors:** Parag Deepak Dabir, Peter Ottosen, Søren Høyer, Stephen Hamilton-Dutoit

**Affiliations:** 1 Institute of Pathology, Aarhus University Hospital, Noerrebrogade 44, DK-8000, Aarhus C, Denmark

**Keywords:** AMACR, Antibody cocktail, Basal cells, Cytokeratins, Immunohistochemistry, p63, Prostate carcinoma

## Abstract

**Background:**

Immunohistochemistry using antibody cocktails against basal cell specific and cancer-associated markers is important in the diagnosis of prostate carcinoma in needle biopsies. We compared the usefulness for detecting prostate carcinoma of a three-marker cocktail of antibodies to α-methylacyl-CoA racemase (AMACR), p63 and cytokeratin (CK) 5 with a traditional two-marker cocktail of AMACR and p63.

**Methods:**

Sixty-six prostate needle biopsies were analysed prospectively. Serial sections were immunostained with the two- and three- antibody cocktails. Blinded slides were assessed individually by two pathologists and sensitivity, specificity and kappa statistics were calculated.

**Results:**

Both antibody cocktails contributed to the detection of prostate carcinoma in needle biopsies. There was an acceptable level of agreement between the pathologists for both the cocktails. Sensitivity was similar for one pathologist comparing both the cocktails (76.4% and 75.7%), but was slightly lower comparing the three-antibody with the two-antibody cocktail for the other pathologist (66.6% *vs.* 77.4%, respectively). Higher specificity values of 90.3% were achieved by both pathologists using three-antibody as compared with two-antibody cocktails (68.7% and 71.8%).

**Conclusions:**

Antibody cocktails are important in diagnosing prostate carcinoma in needle biopsies. Adding an extra basal cell marker to the traditional two-antibody cocktail improves the specificity of detecting prostate carcinoma in limited needle biopsy material, and should be considered for routine diagnostic use.

**Virtual slides:**

The virtual slide(s) for this article can be found here: http://www.diagnosticpathology.diagnomx.eu/vs/2492231327330327

## Background

Prostate cancer is globally the second most frequently diagnosed cancer and the sixth leading cause of cancer death in males, accounting for 14% (903,500) of new cancer cases and 6% (258,400) of cancer deaths in males in 2008 [1]. Incidence rates vary by more than 25-fold worldwide, with the highest rates recorded primarily in the developed countries of Oceania, Europe, and North America, largely because of the widespread use of prostate-specific antigen (PSA) testing and subsequent prostate biopsy in these regions [1].

Histological diagnosis of prostatic cancer is usually based on histological evaluation of prostatic needle biopsies. This can be challenging, particularly when the malignant tissue is limited and is admixed with benign prostatic glands, or because of the presence of benign mimickers of malignancy such as atypical adenomatous hyperplasia (adenosis), atrophy, basal cell hyperplasia, nephrogenic adenoma, seminal vesicles or Cowpers glands [2-4]. In this setting, immunohistochemistry may contribute valuable differential diagnostic information and is used routinely in many pathology laboratories. Whilst a wide variety of immunohistochemical markers have been proposed for this purpose, antibodies against two classes of prostatic biomarkers are most commonly used; firstly, basal epithelial cell-specific markers and secondly, prostate carcinoma-specific markers.

It is widely accepted that absence of basal cells is an important histological criterion for prostate carcinoma. Thus, lack of basal cell staining provides immunohistochemical support for a malignant diagnosis in atypical prostatic lesions [3]. The most commonly used basal cell markers are the high-molecular-weight cytokeratins (such as 34βE12, cytokeratin (CK) 5, CK5/6 and CK14) and p63 [2]. CK 5 is an intermediate-sized cytokeratin that is typically expressed in the basal cells of benign prostate glands, where it shows continuous cytoplasmic staining of the deep layer of the prostate epithelium [5]. p63 is selectively expressed in the basal cell compartment of various epithelial tissues and serves as a sensitive immunohistological target for identifying the nuclei of basal cells in benign prostatic lesions [3].

Several molecules have been proposed as positive immunohistological markers of prostatic carcinoma, including α-methylacyl coenzyme A racemase (AMACR or P504S) and Prostate Tumour Overexpressed-1 (PTOV1) [6]. Of these, the most widely used is AMACR, which is expressed in 80% - 100% of prostatic adenocarcinomas [7]. However, staining for AMACR alone is of limited value as a positive cancer marker, since it is often also expressed in high grade prostatic intraepithelial neoplasia (PIN), in atypical adenomatous hyperplasia (adenosis) and even in atrophic or benign glands [3,7,8]. However, combined staining for a basal cell marker and for AMACR (usually in the form of a two-antibody cocktail) has proved to be an useful immunohistochemical tool for evaluating difficult prostate needle biopsies [9-11] and this type of double staining is widely used in the routine diagnostic setting. For example, in our laboratory we use routinely a two-marker cocktail containing an antibody against p63 (our principal basal cell marker) and an antibody against AMACR, as an immunohistochemical supplement to standard haematoxylin and eosin (H&E) staining when assessing difficult prostatic needle biopsies.

Previous studies have suggested that two basal cell markers may be stained together with AMACR in a triple-antibody cocktail, further improving the ability to recognise limited prostatic carcinoma foci [3,9]. This prompted us to include an antibody to CK5 as an additional basal cell-specific marker to p63 in our routine immunohistological cocktail, and to evaluate these three (AMACR/p63/CK5) and two-marker (AMACR/p63) combinations in a diagnostic setting.

## Methods

Sixty-six prostate needle biopsies received prospectively between January 2011 and June 2011 at the Institute of Pathology, Aarhus University Hospital, Denmark were included in this study. Following standard diagnostic H&E staining, two parallel 4 μm serial paraffin sections were cut from the biopsies for immunostaining with either our traditional two-marker antibody cocktail (AMACR and p63) or the three-marker combination (AMACR, p63 and CK5).

### Immunohistochemistry

The immunohistochemical stains were optimized and tested for specificity using prostate controls including areas of normal tissue, and both benign and malignant disease (data not shown). In the studies, internal controls were present in most biopsies examined. External positive tissue controls were included in each round of stains.

#### Traditional two-marker cocktail

Deparaffinized sections for the two-marker combination were stained in a standard Ventana BenchMark XT automatic stainer using *ultra*View Universal DAB Detection Kit (Ventana Medical Systems, Tucson, AZ), according to the manufacturer. In brief, sections were demasked with Cell Conditioner buffer for 8 minutes and endogenous peroxidase was blocked with *ultra*View inhibitor. The two-marker cocktail containing primary antibodies to AMACR (monoclonal rabbit anti-AMACR, clone 13 H4, Dako, Glostrup, Denmark; dilution 1:100) and p63 (monoclonal mouse anti-human p63, clone 4A4, Dako; dilution 1:300) was applied for 30 minutes. Sections were then incubated with *ultra*View Horse Radish Peroxidase (HRP) Multimer, containing a mixture of HRP-labelled goat anti-mouse and anti-rabbit antibodies. Bound antibodies were visualized by incubation in *ultra*View hydrogen peroxide substrate and DAB chromogen. Positive signals result in a brown colour reaction in the nuclei of benign basal epithelial cells (p63) and in the cytoplasm of malignant prostate epithelial cell cytoplasm (AMACR).

#### Novel three-marker cocktail

Epitope retrieval was achieved by incubating deparaffinised sections in TEG buffer, pH 9 in a microwave at 800 Watts for 8 minutes. Sections were stained on a LabVision Autostainer (Lab Vision, Runcorn, UK). The following primary antibodies were used in a triple-cocktail, applied for 40 minutes: CK5 (rabbit monoclonal anti-CK5, clone EP1601Y, Epitomics, Catalog nr. 1988–1; dilution 1:400), p63 (mouse monoclonal anti-p63, clone 4A4, Dako, code M7247; dilution 1:23) and AMACR (mouse monoclonal anti-AMACR, clone (2A10F3): sc-81710, Santa Crus Biotechnology; dilution 1:25). Bound antibody signals were detected by incubation in polymers for 30 minutes; first with a horse radish peroxidise (HRP) conjugated polymer against the mouse antibodies and then with an alkaline phosphatase (AP) conjugated polymer against the rabbit antibody. Signals were developed using separate chromogens, DAB for the HRP-linked polymer (brown) and Permanent Red for the AP-linked polymer (red). Thus, benign basal prostatic epithelial cells were stained brown in their nuclei (p63) and red in their cytoplasm (CK5), while malignant prostatic carcinoma cells were stained brown in their cytoplasm (AMACR).

The immunostained slides were blinded and then evaluated separately by two pathologists, the first an experienced senior uropathologist, and the second a less experienced junior pathologist. Immunostained slides were evaluated independently of the H&E stained sections. Positive immunohistochemical staining was defined as clear, discrete staining of either the nucleus (p63) or the cytoplasm (CK 5 and AMACR). The two- and three-marker antibody cocktail stains for each biopsy were recorded as giving one of three possible results: 1. *benign*, 2. *intra-epithelial neoplasia* or 3. *malignant*.

Specificity and sensitivity values were calculated for both the two- and three-marker cocktails for each pathologist, compared with a consensus diagnosis based on final evaluation of the H&E and all immunostains. When calculating specificity and sensitivity, results were designated into one of two categories: 1. *test negative/condition absent* (including all biopsies scored as *benign*) or *test positive/condition present* (including all biopsies scored as either *intra-epithelial neoplasia* or *malignant*). Kappa statistics were calculated for each pathologist using the two- and three-marker cocktails for each of the three categories *benign*, *intra-epithelial neoplasia* and *malignant*.

The study was conducted with the ethical board approval from the relevant authorities in Denmark.

## Results

Final diagnoses are not shown in detail, but included a range of benign prostatic disease (32 biopsies), intra-epithelial neoplasia (4 biopsies) and prostatic carcinomas (30 biopsies). All carcinomas were of usual acinar type.

### Immunohistochemistry

#### Traditional two-marker cocktail

**Figure 1:**
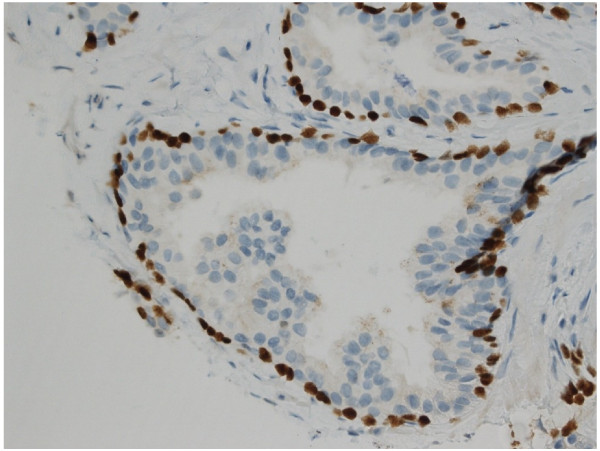
**Immunohistochemical staining of a needle biopsy containing normal prostate tissue with the two-marker cocktail.** Benign basal epithelial cells show dark brown nuclear staining for p63. There is no positive staining for AMACR (x 200).

**Figure 2:**
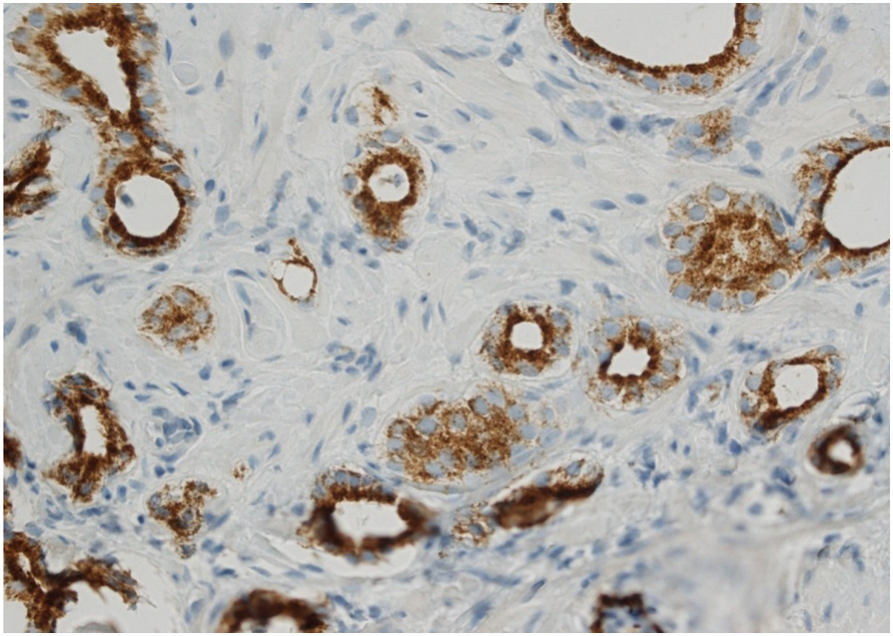
**Immunohistochemical staining of a needle biopsy containing prostatic carcinoma with the two-marker cocktail.** Malignant glands show brown granular cytoplasmic staining pattern for AMACR. Absent nuclear p63 signal indicates loss of basal epithelia cells (x 100).

Benign prostatic glandular tissue showed strong dark brown nuclear staining for p63 in basal cells without any cytoplasmic AMACR staining in the glands [Figure 1]. In contrast, areas of prostatic adenocarcinoma showed absent nuclear p63 staining due to loss of basal epithelial cells, together with the gain of brown granular cytoplasmic staining indicating expression of AMACR in malignant glands [Figure 2].

**Figure 3:**
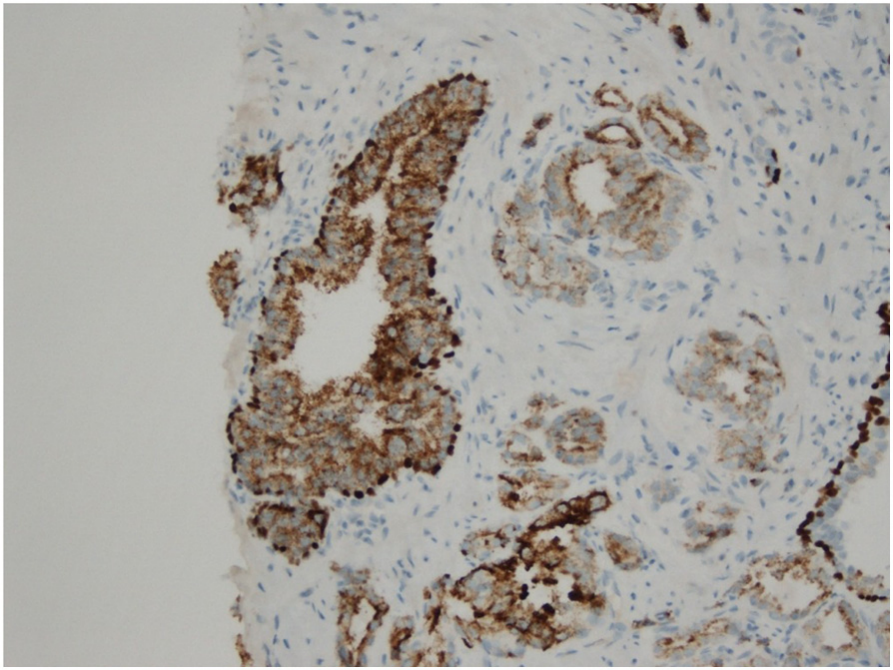
**Immunohistochemical staining of a needle biopsy containing an area of prostatic intra-epithelial neoplasia (PIN) with the two-marker cocktail.** Prostate glands show staining both in the cytoplasm of luminal epithelial cells (brown granular AMACR-positive staining) and in the nuclei of basal epithelial cells (brown nuclear p63-positive staining). This reaction pattern is characteristic for PIN (x 100).

Areas of PIN exhibited both brown cytoplasmic granular AMACR staining in the luminal epithelial cells and dark brown nuclear p63 staining in the associated partially fragmented basal cell layer, thus giving a characteristic positive reaction for both markers in the same glands [Figure 3].

#### Novel three-marker cocktail

**Figure 4:**
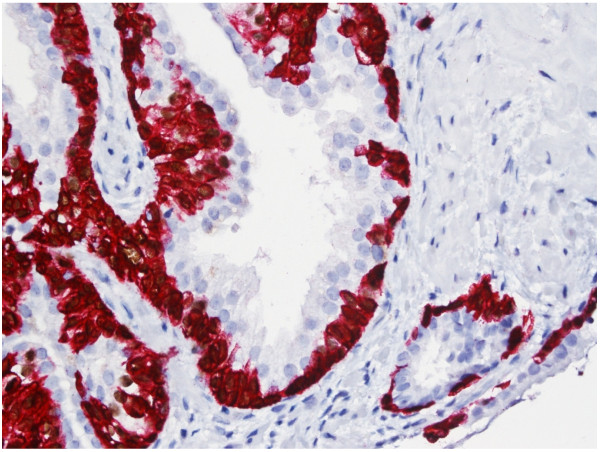
**Immunohistochemical staining of a needle biopsy containing normal prostate tissue with the three-marker cocktail.** Benign basal epithelial cells show both dark brown nuclear staining for p63 and red cytoplasmic staining for CK5. Luminal epithelial cells show no positive staining for AMACR (x 200).

**Figure 5:**
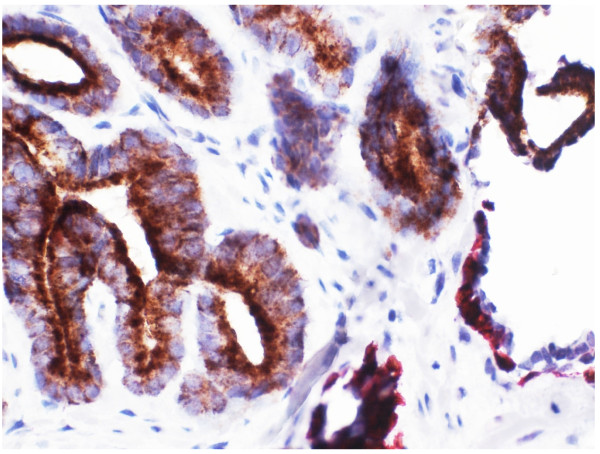
**Immunohistochemical staining of a needle biopsy containing prostatic carcinoma with the three-marker cocktail.** Malignant glands show a brown granular cytoplasmic staining pattern for AMACR with loss of basal epithelia cells (left-hand side). A limited area of benign glandular tissue can be seen on the right-hand side, showing preserved basal epithelial cells with dark brown p63 nuclear staining and red CK5 cytoplasmic staining (x 400).

Benign prostate tissue typically showed both dark brown nuclear (p63) with red cytoplasmic (CK 5) staining in basal cells (sometimes only one basal cell marker was positive), with absent cytoplasmic positivity for AMACR in luminal epithelial cells [Figure 4]. In areas of acinar adenocarcinoma, nuclear and cytoplasmic staining for p63 and CK5, respectively, was absent due to loss of basal cells, whilst the malignant glands showed gain of brown cytoplasmic granular staining indicating expression of AMACR [Figure 5].

**Figure 6:**
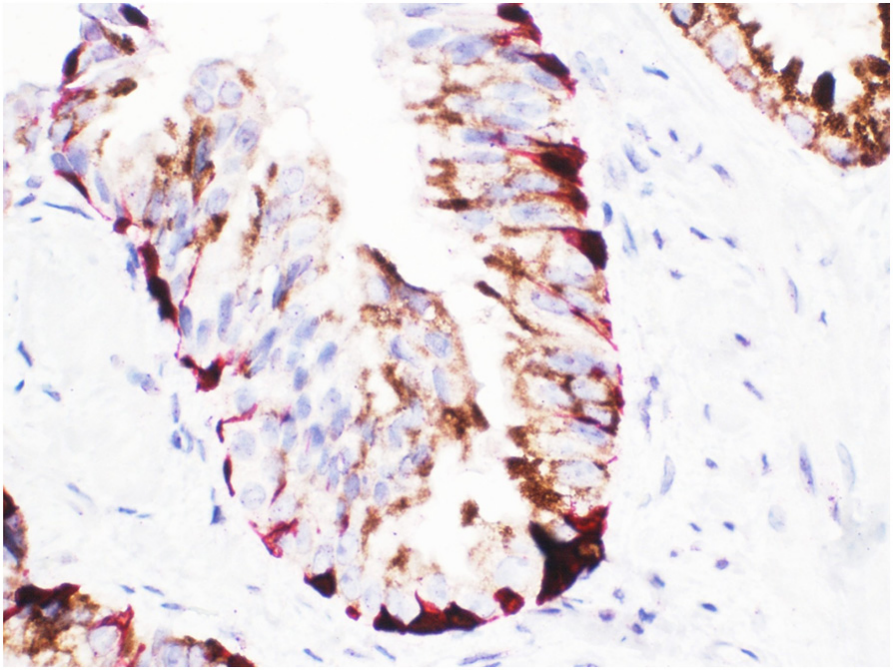
**Immunohistochemical staining of a needle biopsy containing an area of prostatic intra-epithelial neoplasia (PIN) with the three-marker cocktail.** Prostate glands show staining in both the cytoplasm of luminal epithelial cells (brown granular AMACR-positive) and in the basal epithelial cells (brown nuclear p63-positive as well as red cytoplasm CK5-positive), characteristic for PIN (x 400).

Areas of PIN showed both brown cytoplasmic granular AMACR staining in the luminal cells and dark brown nuclear (p63) and/or red cytoplasmic (CK 5) staining in partially preserved glandular basal epithelial cells [Figure 6].

### Comparative evaluation of immunohistochemical cocktails

Both observers found that evaluation of the immunohistological stains with the two cocktails was quick and easy to perform. The pathologists had similar kappa values assessing the biopsies, both using the two-marker (κ = 0.6202) and three-marker (κ = 0.6349) cocktails.

For the junior pathologist, sensitivity and specificity values were 75.7% and 71.8%, respectively, using the two-marker cocktail and 76.4% and 90.3%, respectively, using the three-marker cocktail. Corresponding sensitivity and specificity values for the senior pathologist were 77.4% and 68.7%, respectively using the two-marker cocktail, and 66.6% and 90.3%, respectively with the three-marker combination.

## Discussion

Immunohistochemistry for basal cell-specific and cancer-associated markers is valuable in the histopathological diagnosis of prostate carcinoma in needle biopsies [2,3]. For example, positive AMACR staining helps identify areas of carcinoma, whilst high molecular-weight CKs and the p63 transcription factor aid the search for normal basal cells that are typically absent in malignant prostatic glands. However, using single markers to diagnose prostate carcinoma is of limited use. For example, AMACR may also be expressed in high grade PIN, in atypical adenomatous hyperplasia (adenosis) and even in atrophic or benign glands [3,7]. Moreover, the distribution of basal cells can be patchy in both normal glands and in some benign lesions that mimic prostate cancer, such as areas of atrophy, post-atrophic hyperplasia, and atypical adenomatous hyperplasia (adenosis) [7]. Furthermore, a diagnosis that is reliant on the absence of an immunohistochemical reaction is inherently less reliable. Indeed, a variety of technical problems, including excessive formalin fixation [12], may lead to false negative staining of normal basal cells.

The importance of these immunohistochemical and technical pitfalls in interfering with accurate diagnosis can be reduced by combined staining of prostate biopsies for both positive and negative cancer markers using antibody cocktails. Typically, these cocktails include AMACR together with either an antibody to high molecule-weight CK or to p63. Study by Trpkov et al. demonstrated that CK5/6 is an excellent and dependable basal cell marker when used in combination with AMACR; and CK 5/6 exhibited excellent specificity for prostate cancer, which uniformly lacked CK5/6 staining [5] . Some workers have gone further by including an additional marker for basal cells in three-marker cocktails, in order to try to improve the precision of prostate carcinoma diagnosis in limited biopsy material. Thus, Jiang and co-workers used immunohistochemistry with a triple-antibody cocktail (containing antibodies to AMACR, high molecule-weight 34βE12, and p63) to identify small, focal prostate carcinomas with high sensitivity and complete specificity [9]. Similarly, Ng and colleagues used the same triple-cocktail in a tissue microarray study to identify prostate carcinoma with improved sensitivity (93.8%) and specificity (100%), compared with using the three antibodies individually [3].

Our study supports these previous findings. In addition, we show for the first time that antibody to the high molecular-weight CK5 can be successfully incorporated in triple cocktails together with AMACR and p63. CK5 is widely used as a robust immunohistochemical marker for, amongst other things, basal epithelial cells. Thus, antibodies to CK5 are widely available as routine markers in diagnostic pathology laboratories, and can easily be included in immunohistochemical protocols using automatic staining machines. Although high molecular-weight 34βE12 is included in antibody cocktails for diagnosing prostate carcinoma, there are potential drawbacks associated with the use of this marker. For example, 34βE12 antibody reacts with a wide variety of CKs including not only CK1, CK5, CK10 and CK14, but also an undefined CK that may stain at least some complex epithelia. Thus, 34βE12 may stain breast secretory cells and ductal carcinoma in situ of the breast (which are negative with CK5 antibody), hampering the use of the antibody for the identification of CK5 and myoepithelial cells in the breast [13]. Although this type of reaction has not been reported as occurring in the prostate, any degree of uncertainty about the specificity of a diagnostic antibody is worrying. Similarly p63 when used individually demonstrated loss of immunostaining intensity in stored slides and showed spurious cytoplasmic staining of the luminal cells when used in low dilutions [5].

In our study, similar kappa values were found for both the two-marker and the three-marker cocktails when used by both pathologists, indicating fairly good agreement between them for both cocktails. Both pathologists were equally comfortable evaluating the results of both antibody combinations. In the case of the three-marker cocktail, usage of red staining for CK5 and brown staining for both p63 and AMACR proved to be technically feasible as well as provided a good contrast for interpretation.

Both observers had similar sensitivity levels for correctly assigning biopsies to the diagnostic groups. Comparing use of the three-antibody with the two-antibody cocktail, diagnostic sensitivity was marginally improved for the junior pathologist (76.4% *vs* 75.7%, respectively), but was slightly lower for the senior pathologist (66.6% *vs* 77.4%, respectively). Although the explanation for this is not clear, one likely possibility is that the senior pathologist’s long experience with interpreting stains produced with two-marker cocktails resulted in a paradoxical loss of sensitivity when switching to a novel immunohistochemical stain. Nonetheless, both pathologists showed a marked improvement in the specificity of their various diagnoses comparing the three-antibody with the two-antibody cocktails (90.3% *vs* 71.8% specificity, respectively, for the junior pathologist and 90.3% *vs* 68.7% specificity, respectively, for the senior pathologist). The reasons for this lower false negativity for the basal cell markers in our study could be derived from previous studies stating that CK 5/6 is preserved in the basal cells of non-malignant glands that are cauterized, crushed or distorted for various reasons including laboratory procedures, testifying it’s robustness and reliability [5]. These findings are also comparable to the study by Ng et al.; where 34βE12 was used and CK 5 was one of the several high molecular weight keratins detected by antibody to 34βE12 [3].

## Conclusions

Our study shows that CK5 is an useful supplementary marker for identifying basal cells in prostate needle biopsies. Using CK5 in a three-antibody cocktail markedly improves diagnostic specificity compared with a traditional two-antibody immunohistochemical cocktail. Whilst the findings from our small-sized study should be confirmed in a larger independent study, our results suggest that a three-marker cocktail containing antibodies to AMACR, p63 and CK5 should be considered for routine application in the evaluation of prostatic carcinoma in limited prostatic needle biopsies.

## Competing interests

The authors declare that they have no competing interests.

## Authors’ contributions

PDD and SHD designed the study. PDD and PO performed the immunohistochemical evaluation. PO and SH participated in the design and coordination. PDD and SHD performed the statistical analysis, and drafted the manuscript. PO and SH contributed to improving the draft of the manuscript. All authors have read and approved the final manuscript.
